# Determinants of preterm birth among mothers who gave birth at public hospitals in the Amhara region, Ethiopia: A case-control study

**DOI:** 10.1371/journal.pone.0225060

**Published:** 2019-11-11

**Authors:** Abay Woday, Muluken Dessalegn Muluneh, Samiha Sherif

**Affiliations:** 1 Samara University, College of Medical and Health Sciences, Department of Public Health, Samara, Ethiopia; 2 Amref Health Africa in Ethiopia, Research, Monitoring and Evaluation, Addis Ababa, Ethiopia; 3 Western Sydney University, Sydney, Australia; 4 Amref Health Africa in Ethiopia, Business development, Addis Ababa, Ethiopia; Sefako Makgatho Health Sciences University, SOUTH AFRICA

## Abstract

**Background:**

Preterm birth (PTB) is a public health issue worldwide. In developing nations, like Ethiopia, PTB is under reported and underestimated. However, it is the leading cause of neonatal and under-five mortality in Ethiopia. Besides, limited and non-comparative research studies to date has been conducted in the country to address the prevalence of PTB. Therefore, this study aims to determine predictors of PTB.

**Methods:**

Hospital-based unmatched case control study was employed on a sample of 139 cases and 278 controls from October 2017 to December 2017 in the Amhara region, Ethiopia. The cases and controls were proportionally allocated in each hospital based on the last one-year case flows. As soon as a case was identified, the respective two controls were enrolled until the required sample size was satisfied. The outcome variable was measured by using either last menstrual period (LMP), early ultrasound result, or Ballard maturity examination. Face-to-face interviews were conducted using a standardized, structured, and pre-tested questionnaire to collect data. The collected data was entered into Epi-data and exported into SPSS for analysis. Independent variables with p-values < 0.25 in the bivariate analysis were entered into multivariable logistic regression models with forward logistic regressions method to control the influence of covariates. Ethical clearance was ensured.

**Results:**

A total of 134 cases and 268 controls participated with a response rate of 96.4%. After adjusting for covariates, the following variables were associated with PTB: residing in rural areas [AOR = 2.99: 95% CI 1.19, 7.48], low maternal age [AOR = 3.47: 95% CI 1.11, 10.83], being illiterate [AOR = 4.56: 95% CI 1.11,8.62], short birth spacing [AOR = 2.48: 95% CI 1.07, 5.75], no antenatal care visits for this index pregnancy [AOR = 10.78: 95% CI 4.43, 26.25], having a history of previous adverse birth outcomes [AOR = 3.47: 95% CI 1.51, 8.02], and exposure to medical problems during pregnancy [AOR = 13.94: 95% CI 4.39, 24.27].

**Conclusion:**

The study revealed maternal sociodemographic factors, short birth space, lack of antenatal care, exposure to previous adverse birth outcomes and facing medical illnesses during pregnancy were the predictors of PTB. Therefore, inclusive preventive and control interventions should be developed at regional, zonal and district levels to reduce the burden of PTB among women resided in rural areas such as integrating antenatal care services into the existing health extension packages. Study results suggest increasing the awareness of PTB, contraceptive utilization and counseling to enhance birth spacing, antenatal care visits, and accessibility to services among women in Ethiopia should be given due attention. Health care providers should focus on mothers with previous adverse birth outcomes and those exposed to medical problems during pregnancy. Additional community based longitudinal studies supplemented with qualitative methods are recommended.

## Background

The World Health Organization (WHO) defines preterm birth (PTB) as “a birth which happened before 37 completed weeks of gestations” [1, 2]. Globally, more than 15 million babies are born preterm each year. Of these births, 60 to 85% are concentrated in Africa and South Asia. Besides, the prevalence of PTB ranges between 5% and 18% in the world. In the poorest countries, on average, 12% of babies are born premature compared with 9% in higher-income countries [3–5]. In Ethiopia, the prevalence of PTB ranges from 4.4% to 48.6% [6–10] and its prevalence ranges from 4.4% to 14.3% in the Amhara region [6, 8, 11].

Globally, PTB is the leading cause of newborn deaths within 4 weeks of life and the second cause of under-five mortality, in which 40% of under-five deaths are attributed to prematurity [3, 12–14]. In Ethiopia, prematurity is the first cause of neonatal and the fourth cause of under-five mortality, in which more than 11% of under five deaths and 34% of neonatal deaths are contributed by prematurity [12, 15–17].

Studies conducted across the world identified risk factors associated with preterm birth such as having a previous preterm birth, having a short cervix, Short interval between pregnancies, certain pregnancy related complications (such as multiple pregnancy, pregnancy induced hypertension vaginal bleeding), lack of antenatal care follow-ups, lifestyle factors (such as low pre-pregnancy weight, and substance use during pregnancy) [2, 6, 9, 11, 18].

Survivors of PTB suffer from either long-term or short-term sequeleas, such as breathing difficulties, feeding problems, effects on brain function in later life, cerebral palsy, intellectual and developmental disabilities, visual and hearing impairments, and poor prognosis. Moreover, there are economic burdens of PTB experienced on the individual, family, and societal levels [5, 19, 20].Survival rate of preterm babies is higher in developed nations compared to developing nations. The discrepancy is related to better neonatal care set up and low psychosocial inequality in the developed nations than low-income countries [21, 22].

Globally, including in Ethiopia, various strategies have been attempted in the past several decades to prevent and improve the care of PTBs [14, 23–25]. The literature suggests care strategies for improving outcomes related to PTB such as antenatal corticosteroid use, antibiotics, kangaroo mother care, immediate intensive care, and long-term complex health services. The global community has committed to the reduction of PTBs by aligning initiatives with Sustainable Development Goals (SDGs) and participating in Every Women and Every Child initiatives [26–28]. The government of Ethiopia has also demonstrated commitment to improve the care of newborns and to reduce the burden of PTBs through the implementation of high impact life-saving neonatal interventions in its hospitals. However, the burden of PTB in Ethiopia remains rampant and prevention and control strategies have been given minimal attention in the country.

Existing researches conducted uponPTB were in developed countries. However, limited prevalence studies have been conducted in Ethiopia. Besides, these studies are inconclusive in determining predictors of PTB. Therefore, this study has been designed to be context specific and aims to identify the main predictors of PTB in the Amhara Region.

## Methods and materials

### Study setting and participants

The study was conducted from 1^st^ October 2017 to 1^st^ December 2017 in three randomly selected government referral hospitals: Dessie, Debre Birhan, and Bahir Dar Felege-hiwot referral hospitals located in the Amhara Regional State, Ethiopia. The region has seven referral hospitals, which serve an estimated 22 million individuals.

A hospital-based unmatched case-control study design was conducted to assess the determinants of PTB. Mothers who gave single live births between 28 and 36 completed weeks of gestation during the study period were included as cases and mothers who gave single live births at 37 and above completed weeks of gestation during the study period were included as controls. However, women with induced termination of pregnancy for medical reasons, women giving birth before 28 weeks of gestation, and mothers who were seriously ill during the study period were not included in this study. The gestational age used to determine both the cases and controls were based on either last menstrual period (LMP), early ultrasound result, or Ballard maturity examination.

Sample size was calculated by using Epi info statistical software by considering a double population proportions formula. The researchers had taken the proportion of exposure to any substance (substance use refers to whether a woman had an experience with one or a combination of alcohol, chewing khat, cigarette smoking) during pregnancy among controls (21%) and cases (34.8%) [7]. By considering the basic assumption of 80% power (Zβ = 0.84), 95% CI (Z_a/2_ = 1.96), and r = 2, the research team then calculated the sample size as 121 cases and 242 controls. The assumption that mothers may not give consent following labor due to adverse birth outcomes, exhaustion, or pain led investigators to add 15% to compensate. As a result, the total sample size was 139 cases and 278 controls.

The three hospitals had provided services for a total of 1,080 PTBs within a two-month period in the previous year (2016–2017). The cases were proportionally allocated to the randomly selected referral hospitals (i.e. Dessie = 36 cases, Debre Birhan = 32 cases and Bahir Dar Felege-hiwot hospital = 71 cases). The data collectors assigned to each hospital delivery ward interviewed the eligible case and the consecutive two controls until the required sample size was met. The mothers were interviewed within four to six hours of delivery.

### Data collection tool and procedures

The questionnaire used was adapted and modified from the Ethiopian Demographic and Health Survey [29]. The questionnaire consisted of socio-demographic characteristics, obstetric, medical, and behavioral questions relevant to the experiences of mothers during this index pregnancy (S1 Questionnaire).

The data was collected using face-to-face interviews guided by a standardized, structured, and pre-tested interviewer administered questionnaire. The same interviewer was used to interview both cases and the respective two controls. The outcome variable was attributed to women whose medical records indicated a physician or midwife diagnosis of spontaneous labor onset (with or without intact fetal membranes) and delivery between 28 and 36 completed weeks of gestation. The gestational age (GA) was measured using either LMP, which is found to be a more reliable measure of GA in a low-resource setting [11, 30], early ultrasound result, or Ballard maturity examination within four to six hours of birth. Mothers were interviewed in private rooms to ensure their privacy and to encourage participation. The face-t-face interview was conducted by six trained midwives or nurses who work in the labor wards of each of the selected hospitals.

The measurement tool was translated into the local language (Amharic), then back to English by an independent translator. This method of back-translation was used to maintain accuracy. Pre-testing of the questionnaire was conducted with 5% (21 participants) of mothers who delivered across the three study hospitals. Three days of training was provided for data collectors and the supervisors.

### Data analysis and management

The data was checked for completeness and errors. Then, data was entered into Epi data version 3.1 and cleaned again after data entry. The data was exported and analyzed using SPSS version 20. Before beginning analysis, the research team checked the assumptions, the nature of the variables, frequencies, outliers, and recoded the variables. The descriptive statistics were presented by text, frequency tables, mean with SD, and percentages.

Binary logistic regression analysis was done to evaluate the association of PTB with each pregnancy related factor separately. Variables with a p-value <0.25 were entered into the multivariable logistic regression models. Multivariable logistic regression analysis was performed using the forward Likelihood Ratio method to control for potential confounding variables. Model fitness was tested with Hosmer-Lemeshow goodness of fit test and omnibus tests of model coefficients. Furthermore, correlation between the independent variables was assessed to test multi-collinearity. Finally, the strength of association was measured by both crude and adjusted odds ratios with a 95% confidence interval (CI) for exposure variables and the outcome variable (PTB). Statistical significance level was declared at p-value < 0.05.

## Results

Of a total of 139 cases and 278 controls selected, 134 cases and 268 controls were included, indicating a response rate of 97.1%. Mothers in both the cases and controls had a similar mean age (27.12 years) ± 5.25 SD. A higher proportion of mothers in the case group resided in rural areas compared to mothers in the control groups (60.4% and 31.7% respectively). When comparing the highest completed educational level attended by mothers, a higher proportion of mothers in the the case group did not attended any formal education compared to controls (44.8% and 17.9% respectively) **(Table 1).**

**Table 1:** Socio-demographic characteristics of participants who gave birth at public referral hospitals, Amhara region, Ethiopia, 2018 (n = 134 cases and 268 controls).

Predictor Variables		Cases	Controls	Statistics (X^2^), P-value
		N (%)	N (%)	
Age				
	15–24	38 (28.4)	81 (30.2)	X^2^ = 1.58, P>0.05
	25–34	78 (58.2)	162 (60.4)	
	≥35	18 (13.4)	25 (9.3)	
Residence				
	Urban	53 (39.6)	183 (68.3)	X^2^ = 30.42, P<0.001
	Rural	81 (60.4)	85 (31.7)	
Education level of mother				
	Illiterate	60 (44.8)	48 (17.9)	X^2^ = 32.99, P<0.001
	Primary	35 (26.1)	106 (39.6)	
	Secondary	21 (15.7)	66 (24.6)	
	Tertiary	18 (13.4)	48 (17.9)	
Main occupation of mother				
	Housewife	72 (53.7)	135(50.4)	X^2^ = 1.26, P>0.05
	Farmer	24 (17.9)	45 (16.8)	
	Merchant	14 (10.4)	27 (10.1)	
	Others^+^	24 (17.9)	61 (22.8)	
Total family size				
	< 5	88 (65.7%)	192(71.6%)	X^2^ = 1.51, P>0.05
	≥ 5	46 (34.3%)	76 (28.4%)	

^***+***^ include student, daily labor, and self-employee.

### The obstetric history and medical conditions of participants

A higher proportion of mothers with cases had short spacing between births compared to mothers in the control group. However, a lower proportion of mothers with cases had at least four or more ANC visits compared to mothers in the control group. Mothers with cases had nearly double the exposure history of previous adverse birth outcomes (i.e. previous PTB, LBW (low birth weight), stillbirth and abortion) compared to mothers with controls (27.6% and 13.8% respectively) **(Table 2).**

**Table 2:** The reproductive health and medical conditions of mothers who gave birth at public referral hospitals, Amhara region, Ethiopia, 2018.

Predictor variables		Cases	Controls	Statistics (X^2^), P-value
		N (%)	N (%)	
Nutritional status of mother (in MUAC)				
	SAM	7 (5.2)	3 (1.1)	X^2^ = 7.14, P<0.05
	Moderate	12 (9.0)	34 (12.7)	
	Normal (≥ 23 cm)	115 (85.8)	231 (86.2)	
Birth interval for this pregnancy				
	< 2 years	26(33.8)	23 (17.8)	X^2^ = 6.75, P<0.01
	≥ 2 years	51 (66.2)	106 (82.2)	
ANC follow up				
	Yes	122 (91.0)	262 (97.8)	X^2^ = 9.42, P<0.01
	No	12 (9.0)	6 (2.2)	
Gestational Age at ANC initiation (n = 122)				
	<4 months	45 (36.9)	127 (48.5)	X^2^ = 4.52, P<0.05
	≥ 4 months	77 (63.1)	135 (51.5)	
Number of ANC visits				
	< 4 visits	88 (65.7)	61 (22.8)	X^2^ = 70.51, P<0.001
	≥ 4 visits	46 (34.3)	207 (77.2)	
Hypertension during pregnancy				
	Yes	29 (21.6)	28 (10.4)	X^2^ = 9.19, P<0.01
	No	105 (78.4)	240 (89.6)	
HIV Status of the mother				
	Reactive	4 (3.0)	12 (4.5)	X^2^ = 0.52, P>0.05
	Non-reactive	130 (97.0)	256 (95.5)	
Medical problems during this pregnancy (PROM, DM, Renal, UTI)				
	Yes	52 (38.8)	21 (7.8)	X^2^ = 57.6,P<0.001
	No	82 (61.2)	247 (92.2)	
Previous Adverse birth outcomes				
	Yes	37 (27.6)	37 (13.8)	X^2^ = 11.33,P<0.01
	No	97 (72.4)	231 (86.2)	

### Substance use during pregnancy

Mothers with PTBs have consumed more alcohol containing drinks compared to their counterparts (52.2% and 40.3% respectively). A higher proportion of fathers of cases have consumed more alcohol than control groups [57.3% and 39.9% respectively] **(Table 3).**

**Table 3:** The substance use of mothers who gave birth at public referral hospitals, Amhara region, Ethiopia, 2018.

Predictor variables		Cases	Controls	Statistics (X2), P-value
		N (%)	N (%)	
Mother chewing khat during pregnancy				
	Yes	25 (18.7)	50 (18.7)	X^2^ = 0.01, P>0.05
	No	109 (81.3)	218 (81.3)	
Frequency of khat intake				
	Daily	2 (11.1)	1 (1.1)	X^2^ = 7.04, P<0.05
	At least once Per week	12 (48.1)	37 (40.7)	
	Occasionally^*^	11 (40.7)	53 (58.2)	
Mother drink Alcohol during pregnancy				
	Yes	70 (52.2)	108 (40.3)	X^2^ = 5.16, P<0.05
	No	64 (47.8)	160 (59.7)	
Frequency of alcohol intake				
	Daily	4 (5.7)	4 (3.7)	X^2^ = 14.99, P<0.01
	At least once Per week	28 (40.0)	29 (26.6)	
	At least once per fortnight	24 (34.3)	68 (62.4)	
	Occasionally^*^	14 (20.0)	8 (7.3)	
Mother smoke cigarette				
	Yes	2 (1.5)	1 (0.4)	X^2^ = 1.51, p>0.05
	No	132 (98.5)	267 (99.6)	
Husband currently chewing khat				
	Yes	38 (28.4)	105 (39.2)	X^2^ = 4.56, P<0.05
	No	96 (71.6)	163 (60.8)	
Husband currently smoke cigarette				
	Yes	5 (3.7)	11 (4.1)	X^2^ = 0.033, P>0.05
	No	129 (96.3)	257 (95.9)	
Husband currently drink Alcohol				
	Yes	77 (57.5)	107 (39.9)	X^2^ = 11.06, P<0.01
	No	57 (42.5)	161 (60.1)	

* taking substance at holy day time or for entertainment purposes

### Determinants of PTB

Bivariate logistic regression analysis was performed using odds ratios (OR) and 95%CI. The predictor variables with p-value less than 0.25 in the bivariatelogistic regression analysis were entered into the multivariable logistic regressionanalysis model to control the influence of potential confounding variables. The correlation between the independent variables was checked. Moreover, the fitness of the model was also assessed.

After adjusting for covariates, women who resided in rural areas had three times higher odds of PTB compared to women who resided in urban settings [AOR = 2.99: 95% CI 1.19, 7.48]. The likelihood of PTB among women in the age group 25–34 years of age was three times higher compared to women greater or equal to 35 years of age [AOR = 3.47: 95% CI 1.11, 10.83]. Women who were illiterate had five-folds higher odds of PTB compared to women who attended tertiary education [AOR = 4.56: 95% CI 1.11, 8.62]. Women with short birth intervals prior to this index pregnancy (<2 years) had 2.5 times higher odds of PTB compared to women with long birth intervals [AOR = 2.48: 95% CI 1.07, 5.75]. Women who had not had antenatal care visits for this index pregnancy had five-folds higher odds of PTB compared to those who had antenatal care visits [AOR = 5.18: 95% CI 3.43, 16.25].

Women with a history of adverse birth outcomes (i.e. abortion, PTB, low birth weight, stillbirth) prior to this index pregnancy had 4.5 times higher odds of PTB compared to those who did not have a history of adverse birth outcomes [AOR = 4.57: 95% CI 1.51, 8.02]. Moreover, women who experienced medical problems (PROM, UTI, DM and renal diseases) during pregnancy had 14-folds higher odds of PTB compared to those who were not exposed to any medical problems during this index pregnancy [AOR = 13.94: 95% CI 4.39, 24.27] (**Table 4**).

**Table 4:** Determinants of PTB among mothers who gave birth at public hospitals, Amhara region, Ethiopia, 2018.

Predictor variables		Birth outcomes		COR (95% CI)	AOR (95% CI)
		Preterm	Term		
Residence	Urban	53 (39.6)	183 (68.3)	1.00	1.00
	Rural	81 (60.4)	85 (31.7)	3.29 (2.13, 5.06)	2.89 (1.19, 7.48)*
Age in years	15–24	38 (28.4)	81 (30.2)	0.65 (0.32, 1.33)	0.94 (0.19, 4.68)
	25–34	78 (58.2)	162 (60.4)	0.66 (0.34, 1.29)	3.47 (1.11, 10.83)*
	35 and above	18 (13.4)	25 (9.3)	1.00	1.00
Completed educational level	Illiterate	56 (41.8)	31 (11.6)	3.33 (1.72, 6.46)	4.56 (1.11,8.62)*
	Primary school	27 (20.1)	76 (28.4)	0.88 (0.45, 1.71)	2.14 (0,49, 9.28)
	Secondary school	25 (18.7)	97 (36.2)	0.85 (0.41, 1.76)	3.04 (0.65, 14.18)
	Tertiary school	26 (19.4)	64 (23.9)	1.00	1.00
Birth interval	< 2 years	26 (33.8)	23 (17.8)	2.35 (1.22, 4.51	2.48 (1.07, 5.75)*
	> = 2 years	51 (66.2)	106 (82.2)	1.00	1.00
ANC follow up	Yes	122 (91.0)	262 (97.8)	1.00	1.00
	No	12 (9.0)	6 (2.2)	4.29 (1.57, 11.71)	5.18 (3.43, 16.25)**
Previous adverse birth outcome (abortion, PTB, LBW, still birth)	Yes	37 (27.6)	37 (13.8)	2.38 (1.42, 3.98)	4.57 (1.51, 8.02)**
	No	97 (72.4)	231 (86.2)	1.00	1.00
Nutritional status (in MUAC)	SAM	7 (5.2)	3 (1.1)	4.68 (1.19,18.46)	8.75 (0.60, 7.67)
	Moderate	12 (9.0)	34 (12.7)	0.71 (0.35, 1.42)	0.42 (0.11, 1.62)
	Normal	115 (85.8)	231 (86.2)	1.00	1.00
Hypertension during pregnancy	Hypertensive	29 (21.6)	28 (10.4)	2.36 (1.34, 4.17)	0.87 (0.28, 2.70)
	Normal	105 (78.4)	240 (89.6)	1.00	1.00
Alcohol consumption during pregnancy	Yes	70 (52.2)	108 (40.3)	1.62 (1.06, 2.46)	1.16 (0.51, 2.60)
	No	64 (47.8)	160 (59.7)	1.00	1.00
Medical problems during pregnancy (DM, UTI, PROM…)	Yes	52 (38.8)	21 (7.8)	7.46 (4.23, 13.12)	13.94 (4.39, 24.27)***
	**No**	82 (61.2)	247 (92.2)	1.00	1.00

AOR-Adjusted Odds ratio, COR-Crude odds ratio, CI- Confidence Interval,

* = p<0.05,

** = P<0.01,

*** = P<0.001, DM- Diabetes Mellitus, UTI- Urinary Tract Infection, PROM-Premature Rupture of Membrane, MUAC- Mid-Upper Arm Circumference, PTB-Preterm Birth, LBW-Low Birth Weight

## Discussion

The study indicates that the odds of PTB among mothers in rural areas is higher than women in urban areas, which is consistent with a study conducted in Jimma Specialized Teaching Hospital in southwest Ethiopia [7] and a study conducted in Bahir Dar in northwest Ethiopia [9]. This may be linked to poor accessibility, availability, and utilization of health care services among women living in rural communities compared to women living in urban areas. In addition, women resided in a rural community of Ethiopia have less exposure to media channels which is commonly used during health promotion to address pregnancy-related health problems at country level and regional levels.

The study revealed that as the age of the mother increases, the risk of PTB decreases. This finding is supported by a study conducted in Egypt [31] and a longitudinal cohort study done in Canada [32]. This may be because as the age of women increases, their health seeking behavior and awareness regarding pregnancy related health problems will also increase. Moreover, young women are more exposed to many risk behaviors like substance use and less adherence to counseling and education given by their healthcare providers compared to older women. Therefore, the risk of preterm birth decreases when the age of women increases.

Illiterate women had 5 times a greater risk of PTB compared to women attended tertiary level education. This finding is similar to a study done in Iran [21], and case-control study conducted among Egyptian women [31]. This could be explained by illiterate mothers may have lower knowledge regarding the available health services and how to utilize these available services. Thus, these women are prone to preterm births compared to mothers attended tertiary level education.

In this study, women who had no ANC visits had five-folds higher odds of PTB compared to those who had ANC follow-up during this index pregnancy. This result is similar to a study done in public health facilities in Debre Markos town, northwest Ethiopia [11] and a study conducted in Negist Elene Mohammed Memorial General Hospital in Hosanna Town, southwest Ethiopia [33]. This could be because women without ANC follow-up are not aware of available health services and do not receive counseling about the warning signs of risk during pregnancy, risky behaviors, and more. Therefore, these women are at a higher risk of adverse birth outcomes and pregnancy related complications compared to those who have had at least four or more ANC follow-ups.

The odds of PTB among women with short birth interval (less than two year intervals) prior to this index pregnancy was 2.5 times higher compared to women having longer birth intervals. The WHO recommends two-year birth intervals before attempting the next pregnancy in order to reduce the risk of adverse maternal, perinatal, and infant outcomes. This finding is in line with a study done in Bahir Dar Felege-hiwot referral hospital, northwest Ethiopia [9]. This could be because mothers with short birth intervals are not physiologically, physically, economically and psychologically well prepared to endure another pregnancy and childbirth. Thus, they have no optimal time to care themselves and the coming newborn. As a result, these women, are at a higher risk of adverse birth outcomes like PTB compared to women gave birth with WHO recommended optimal birth intervals.

The likelihood of PTB among women with a history of adverse birth outcomes was found to be 4.5 times higher compared to those who did not have a history of adverse birth outcomes. This finding is in line with a study conducted in Jimma, southwest Ethiopia [7], a study conducted in Gondar Hospital, northwest Ethiopia [8], a prospective cohort study done in Tanzania, and a case-control study conducted in west Iran [34]. This could be explained by an increased risk of recurrence in successive pregnancies once a woman has experienced an adverse birth outcome; also, women who have faced adverse birth outcomes in the past may undergo stress due to their negative outcome which is a biologically plausible determinant of PTB.

Women exposed to medical problems during pregnancy had 14-folds higher odds of PTB compared to those who were not exposed to any medical problems during this index pregnancy. This finding is in line with a study done in Jimma, southwest Ethiopia [7], a study done in Gondar, northwest Ethiopia [6], and a study done in Debre Markos town, northwest Ethiopia [11]. Medical problems during or prior to pregnancy may have an impact on the placenta, the uterus, membrane, or the fetus. Generally, they are at a higher risk of birth complications including PTB [11, 35]. Therefore, women who experience medical problems during or prior to pregnancy need urgent attention from health care providers to prevent adverse birth outcomes (i.e. PTB, LBW, stillbirth and abortion).

The study revealed that there is no statistically significant association between alcohol consumption during pregnancy and PTB. The finding is consistent with a prospective study conducted in South Africa which revealed that alcohol consumption did not have a significant effect on the incidence of PTB [36]. However, this finding is not supported by a study conducted in Jimma Specialized Hospital, southwest Ethiopia [7]. The discrepancy can be explained by the under-reporting of alcohol consumption by women in the northern part of Ethiopia due to the fear of stigmatization and sociocultural grounds, and small sample size in the present study compared to the women in Jimma.

### Limitations of the study

Since the study deals with personal and sensitive behaviors, such as substance use during pregnancy, there is a possibility of falsified reporting among mothers, especially given the face-to-face interview modality of data collection. Another limitation of the study is being institution–based study; in Ethiopia institutional delivery is very low and this study may not represent as close to 75% of deliveries which take place at homes.

## Conclusion

The study revealed that maternal sociodemographic factors, short birth space, lack of antenatal care, exposure to previous adverse birth outcomes and experiencing medical illnesses during pregnancy were the main predictors of preterm births.

It is important to design inclusive and new strategies at national regional, zonal, district levels to reduce the burden of PTB among women living in the rural community. The suggested strategies include; integrating the antenatal care services into the existing health extension packages; establishment of comprehensive mobile clinic services to address hard to reach areas. Besides, the regional government should work on accessibility of available health services for the rural community.

Health care providers should give due attention to mothers with previous adverse birth outcomes, those with medical problems during pregnancy, and to provide counseling regarding birth spacing mechanisms. Additional longitudinal and community based studies supplemented with qualitative methods are recommended.

### Ethical issues and consent to participate

Ethical approval was obtained from the Research and Ethics Committee (REC) of the School of Public Health, Addis Ababa University by the letter written on February 23, 2018 and project number 40/2018 for appropriateness and scientific content of the study. The study was conducted in the selected hospitals after permission letters were obtained from the respective health sectors (district health offices). All Participants including 15 years of ages were asked for informed verbal consent before participating in the study. In Ethiopian context, once women get married they have the right to decide on their personal issues including participation in research. They were provided with information regarding the purpose, objective, procedures, potential risks, and benefits of the study; they were also assured of strict confidentiality with regard to information obtained. No personal identifiers were used and verbal consent was taken after explaining the stated risks. There was no denial of health service for refusal to participate in the study. There was immediate linkage to the psychiatric clinic for those in need of counseling during or after interview. Each participant was assured they have the right to refuse, ask questions, and to discontinue at any time. The name or identification card of participants was not disclosed to maintain the confidentiality/anonymity of the data.

## Supporting information

S1 File Questioniare. (DOCX)

## Abbreviations

ANC: Antenatal Care
AOR: Adjusted Odds Ratio
CI: Confidence Interval
COR: Crude Odds Ratio
PTB: Preterm Birth
SPSS: Statistical Package for Social Science
WHO: World Health Organization
