# Supplementary material for: Determinants of preterm birth among mothers who gave birth at public hospitals in the Amhara region, Ethiopia: A case-control study
Source: PLoS One. 2019 Nov 11;14(11):e0225060. doi: 10.1371/journal.pone.0225060 (PMC6844458; doi:10.1371/journal.pone.0225060)
Supplement: S1 File — (DOCX) [file pone.0225060.s001.docx]

# **10. ANNEX**

## Annex I: PARTICIPANT INFORMATION SHEET Dear participants

My name is …… I am here on the behalf of Mr. Abay W., Mr Mulluken D., Samiha S. who are author of this research. They are working this research on the determinants of preterm birth. Prior to this, I will explain information about the study and requesting you to participate in the study.

**Purpose:** To identify the determinants of preterm birth.

**Benefit**: This study will help to identify the main determinants of preterm birth and to improve the health of both mothers and their newborns. Furthermore, to prevent preterm birth and consequence of preterm birth. Mothers with preterm births were linked to the NICUs if they need advanced care.

**Harm**: The study will take time of you and may have a little discomfort while remembering the occasion happening to you but does not have any risk.

**Confidentiality and right of participant:** All the responses given by you and results obtained from you will be kept confidential using coding system whereby no one will have access to your response. Without permission from you and legal body, any part of this study will not be disclosed to third person. You are not expected to give your name or phone number. Your participation in this research is voluntary. You have full right to refuse to a question that you don’t want to answer and withdraw from the participation at any time if you don’t want to continue. There will not be any negative consequence and benefit because of participating and not participating on the study. However, your participation on this study is very important for improvement of both the mothers and their newborns health.

**Duration of the interview:** This interview will take approximately 30 – 40 minutes.
⮚ Do you have any question regarding the aim and content of the interview?
Are you willing to participate on the study?

1. Yes 2) No

If you are willing to participate in this study, please sign the agreement form written on in the informed consent form below.

Annex II: INFORMED CONSENT
It has been read to me in the language I understand. I understand that I can continue or discontinue the interview without any problem. Therefore,

1) I agree to participate

2) I refuse to participate

If the participant agrees to participate, skip to the next page.
If no, skip to the next participant by writing short reasons for refusal below.
………………………………………………………………………………………
Name of investigators: Abay W., Mulluken D. Samiha S.
Principal investigator: Abay Woday Tadesse

Address: Mob +251-9-11-37-68-50
E-mail: [abaywoday@yahoo.com](mailto:abaywoday@yahoo.com)
Supervisor address Tel …………………………….
Date of interview ………………………………….
Time started: ……………………………………….
Time completed …………………………………….
Name of data collector: …………………………………
Checked by:
Supervisor Name: ……………………… Sig……………………

## Annex III: English version Questionnaire:

| Abay W, Mulluken D, Samiha S. Determinants of Preterm Birth Among Mothers Who Gave Birth at Public Hospitals in the Amhara Region, Ethiopia: A Case-Control Study  Contact address of the Principal investigator: [abaywoday@yahoo.com](mailto:abaywoday@yahoo.com)  Cell phone: (+251) 0911376850 |
| --- |

**Identification related information**

Data collector’s Name: ______________________ Signature: ____________Date___________

Supervisor’s Name: ________________________ Signature: ___________________________

Questionnaire Code No: ____________________ Hospital Name: _______________________

**Part I: Now, I am going to ask some questions about your Background (socio-demographic and socioeconomic) information that is very important to the study.**

| ID code | Questions | Code of variables | Skip |
| --- | --- | --- | --- |
|  | How old are you in complete years? | _______ (in completed years) |  |
|  | Where is your place of residence? | 1. Urban  2. Rural |  |
|  | What is your marital status? | 1. married /live together/ 2. divorced 3. widowed 4. single |  |
|  | How old were you when you get your first marriage? | _______ (in completed years) |  |
|  | What is your religion? | 1. Orthodox 2. Muslim 3. Protestant 4. Other(specify)_______ |  |
|  | Do you read and write simple sentences with any language you speak? | 1.Yes  2. No | Skip to Q108 |
|  | What was the highest level of education you have attended? | 1. Grade ………………… 2. Informal (X) ------------ |  |
|  | What is your main Occupation? | 1. Housewife 2. Farmer 3. merchant 4. Government employee 5. NGOs employee 6. Engaging small business/Micro 7. Others (Specify)___________ |  |
|  | What is your total Family size (including extended families)? | ____________(in number) |  |

**Part II: Now, I would like to ask some questions regarding to your obstetrics and medical conditions in the last 9 months (Obstetric and Medical conditions).**

| Code | Questions | Code of variables | Skip |
| --- | --- | --- | --- |
|  | How many times you became pregnant including this pregnancy? | ________(put in number) |  |
|  | How many was the interval or space prior to the current pregnancy? | 1.___________ (In completed months or years)  22. I don’t know |  |
|  | Did you have ANC follow-ups for this pregnancy? | 1. Yes 2. No | Skip to Q209 |
|  | How many was the gestational Age of fetus when you start ANC follow-ups? | 1. ______ (in Weeks)   22. I don’t remember |  |
|  | Where you start your ANC? | 1. Health post 2. Health center 3. Government Hospital 4. Private hospital 5. Private clinic 6. NGOs clinic 7. Other (specify)______________ |  |
|  | How many times you visit health facilities for ANC only? | ________ (put in number)  22.I don’t remember |  |
|  | Have you told as you have any danger symptoms of pregnancy during your ANC visits? | 1. Yes 2. No |  |
|  | If your answer is yes for Q207, Which danger symptoms do you have? (**multiple response possible** | 1. Severe headache 2. Blurred vision 3. Epigastric pain 4. Vaginal bleeding 5. Others (specify)__________ |  |
|  | Have you told as you had any medical problems in the last 9 months related to this pregnancy? | 1. Yes 2. No | Skip to Q211 |
|  | If your answer is yes for Q213, Which medical problems? (**multiple response possible** | 1. DM 2. Renal problem 3. Cardiac problem 4. Liver problem 5. PROM 6. UTI 7. Others (specify)_________ |  |
|  | Do you have History of Adverse birth outcomes before this pregnancy? (Abortion, Low birth weight, Preterm birth, Small for gestational age, Still birth) | 1. Yes 2. No | Skip to Part III |
|  | If your answer is yes for Q210, which one of the followings? (**multiple response possible)** | 1. Preterm birth  2.. Low birth weight  3.. Abortion  4.. Still birth  5. SGA  5.. other (specify)………………… |  |

**Part III: Now, I would like to ask some questions regarding to the lifestyle you have in the last 12 months (Behavioral conditions of both mother and husband).**

| ID | Questions | Code of variables | Skip |
| --- | --- | --- | --- |
|  | Have you ever chewed Khat? | 1. Yes 2. No | Skip to Q304 |
|  | Have you chewed Khat in the last 9 months? | 1. Yes 2. No |  |
|  | How often do you chew khat? | 1. Every day 2. At least once Per week 3. At least once Per fortinight 4. Occationally 5. Not at all |  |
|  | Have you ever smoke cigarette? | 1. Yes 2. No | Skip to Q308 |
|  | Did you smoke cigarette in the last 9 months? | 1. Yes 2. No |  |
|  | How often do you smoke? | 1. Every day 2. At least once Per week 3. At least once Per fortnight 4. Occasionally 5. Not at all |  |
|  | How many cigarettes you smoke per day? | ________(in number) |  |
|  | Did you drink Alcohol in the last 9 months? | 1. Yes 2. No | Skip to Q310 |
|  | How often do you drink alcohol? | 1. Every day 2. At least once Per week 3. At least once Per fortinight 4. Occasionally 5. Not at all |  |
|  | Did your husband chew Khat? | - - - 1. Yes       2. No | Skip to Q312 |
|  | How often does he chew khat? | - - - 1. Every day       2. At least once Per week  1. At least once Per fortinight 2. Occationally 3. Not at all |  |
|  | Does your husband smoke cigarette? | 1. Yes  2. No | Skip to Q314 |
|  | How often does he smoke cigarette? | - - - 1. Every day       2. At least once Per week       3. At least once Per fortinight       4. Occationally       5. Not at all |  |
|  | Does your husband drink Alcohol? | 1.Yes  2. No |  |
|  | If yes Q315, How often does (did) he get drunk? | 1.Every day  2. At least once Per week   - - - 1. At least once Per fortnight       2. Occasionally       3. Not at all |  |

**Part IV: Questions to be filled from medical records or by Measurement**

1. What is your Gestational age at birth? (**based on** **LMNP or see card for physician Diagnosis or see early ultrasound result)** ________ (put In completed weeks)
2. What is the Weight of the new born at birth (**measure or see cards**) __________ (put in grams)
3. What is the Weight of the mother at birth (**measure**) ______ (in grams)
4. What is the height of the mother at birth (**measure**) ______ (**in centimeters**)
5. What is the MUAC of the mother at birth (**measure**) ______ (**in centimeters**)
6. What is the recent or current BP of mother? (Measure or see card?_______//_____mmHg
7. What is the current sero status of mother? (Test or see card?_______(R/NR)

**Thank You!**
